# Leptin status in adolescence is associated with academic performance in high school: a cross-sectional study in a Chilean birth cohort

**DOI:** 10.1136/bmjopen-2015-010972

**Published:** 2016-10-18

**Authors:** Paulina Correa-Burrows, Estela Blanco, Marcela Reyes, Marcela Castillo, Patricio Peirano, Cecilia Algarín, Betsy Lozoff, Sheila Gahagan, Raquel Burrows

**Affiliations:** 1 Institute of Nutrition and Food Technology, University of Chile, Santiago de Chile, Santiago, Chile; 2 Child Development and Community Health Division, University of California San Diego, La Jolla, California, USA; 3 Center for Human Growth and Development, University of Michigan, Ann Arbor, Michigan, USA

**Keywords:** leptin, hyperleptinemia, cognition, academic performance, adolescents

## Abstract

**Objective:**

Leptin is a pleiotropic hormone associated with learning and memory via brain receptors. However, elevated plasma leptin levels may impair cognitive and memory functions. Since individual differences in memory performance affect students’ ability to learn, we aimed to study the relation between leptin status in adolescence and school performance.

**Design and setting:**

We studied 568 adolescents aged 16–17 years from Santiago. A cross-sectional analysis was carried out on a birth cohort conducted in Santiago (Chile).

**Primary and secondary outcome measures:**

We measured serum leptin concentration using an enzyme-linked immunosorbent assay. Cut-offs from the Healthy Lifestyle in Europe by Nutrition in Adolescence (HELENA) Study for 16-year-olds were used to define abnormally high leptin levels (hyperleptinaemia). Academic performance was measured using high-school grades and grade point average (GPA). Data were collected in 2009–2012; data analysis was performed in 2014.

**Results:**

15% of participants had hyperleptinaemia. They had significantly lower school grades and GPA compared with participants with normal leptin levels (eg, GPA mean difference=33.8 points). Leptin levels were negative and significantly correlated with school grades in 9th, 10th and 12th. Similarly, it was negatively correlated with high-school GPA. After controlling for health, sociodemographic and education confounders, the chances of having a performance ≥75th centile in students having hyperleptinaemia were 32% (95% CI 0.19% to 0.89%) that of students having normal serum leptin concentration.

**Conclusions:**

In high school students, abnormally high levels of leptin were associated with poorer academic performance. These findings support the idea of a relationship between leptin and cognition. Further research is needed on the cognitive effects of leptin in younger populations.

**Strengths and limitations of this study**

- This paper is the first to link leptin status in a healthy younger-age human population with functional measures of cognition (high-school grades), aiming at examine this relation in the ‘real’ world.
- Our sample is not representative of the Chilean adolescent population, as it consisted of adolescents from low-to-middle socioeconomic status (SES). However, the prevalence of risk of unhealthy dietary habits and obesity, both of which may lead to abnormally high leptin levels, is higher in these groups.
- We used cut-offs for hyperleptinaemia defined on the basis of statistical criteria, which are the only values described for healthy adolescents. Future studies should use cut-offs based on biological risk.
- We did not consider the mediating effect of other important influences, like the prevalence of neuropsychiatric conditions and learning disorders, which may impact student's academic functioning. Also, information on family structure is lacking in the analysis.
- Since association does not imply causation, future studies should replicate this analysis in other young populations.

## Introduction

Leptin, the protein hormone produced in fat tissue which regulates the amount of fat stored in the body, was originally thought to be involved only in the regulation of food intake and energy balance. Recent evidence shows that leptin also plays a role in physiological courses other than eating behaviour; in fact, it can influence several developmental processes in the immature brain.^1–3^

Leptin receptors are expressed throughout the brain, especially in the hippocampus and various cortical regions. Numerous evidence supports the role of leptin in higher cognitive functions, particularly the ability to boost physiological events underlying hippocampal-dependent learning and memory.^4^ In the hippocampus, leptin facilitates the induction of synaptic plasticity by converting short-term potentiation of synaptic transmission into long-term potentiation (LTP), a process regarded as part of the neurophysiological basis of learning and memory formation.^5^ Impairment of this process is associated with cognitive deficits.^1^ ^6^ ^7^ In the prefrontal cortex, leptin is associated with increased brain-derived neurotrophic factor expression and neurogenesis.^8^ ^9^

If leptin in the physiological range may serve as a cognitive enhancer, elevated plasma leptin levels or hyperleptinaemia may act as a pathophysiological marker for impaired cognitive function due to tissue leptin resistance (LR). The cognitive effects of leptin depend on its ability to cross the blood–brain barrier (BBB) and the functionality of leptin receptors within the hippocampus and other brain regions.^10^ The inability of peripheral leptin to reach the brain is called peripheral LR, while a diminished leptin receptor quantity and impaired signal transduction is known as central LR. In humans, both defects coexist. Also, hyperleptinaemia is a strong indicator of LR.^11^ Ageing, disease such as diabetes and neurodegenerative disorders, and excessive exposure to saturated fats and refined sugars (eg, triglycerides and fructose) have been associated with dysfunctional leptin transport into the brain.^12–15^ On the other hand, obesity has been initially associated with a period of central leptin hypersensitivity, followed by a phase of central LR.^12^ ^16^

Elevated plasma leptin levels have been associated with poorer cognitive outcomes in the middle-aged and elderly population as well as in certain diseases, including diabetes and Alzheimer's.^3^ Although adolescence is an important period for shaping memory, very few studies have approached this topic in younger age groups, and they have mostly used animal models.^17–20^ Aiming to translate knowledge from research to practice and policy, the objective of this study was to assess the association between leptin and cognition in youths in the ‘real’ world by using functional cognitive measures such as school grades and grade point average (GPA). Since leptin modulates the cellular processes underlying hippocampal-dependent learning and memory, and also since memory skills are good predictors of academic outcomes,^21^ ^22^ we hypothesised that abnormally high circulating leptin would affect the ability of adolescents to perform well in school.

## Methods

### Study sample

We studied adolescents aged 16–17 years living in Santiago, Chile, from low-to-middle socioeconomic status (SES), who were part of a birth cohort. Participants were recruited at 4 months from public healthcare facilities in the southeast area of Santiago (n=1791). They were born at term of uncomplicated vaginal births, weighed >3.0 kg and were free of acute or chronic health problems. At 6 months, infants free of iron deficiency anaemia (IDA) (n=1657) were randomly assigned to receive iron supplementation or no added iron (ages 6–12 months). They were assessed for developmental outcomes in infancy, 5, 10 and 15 years.^23^ At 16–17 years, those with complete data in each wave (n=678) were also assessed for obesity risk and the presence of cardiovascular risk factors in a half-day evaluation that included a fasting blood draw.^24^ Of them, n=568 (84% of those participating in the obesity/cardiovascular study) had completed high school (HS) by mid-2014 and met the criteria for this study. Participants and their primary caregiver provided informed and written consent, which was designed following the norms for Human Experimentation, Code of Ethics of the World Medical Association (Declaration of Helsinki, 1995).

### Measures

#### Data collected in the 16–17 years wave

##### Leptin status in adolescence

After a 12 hours overnight fast, a fasting venous blood sample was collected (08:00–9:00). Serum specimens were separated by centrifugation at 3000 rpm for 10 min at 4°C, and stored at −70°C. Serum leptin was measured by a sensitive ELISA (Active Human Leptin ELISA, DSL-10-23100, Diagnostic System, Webster, Texas, USA). The minimum detectable concentration was 0.05 μg/L. The intra-assay and interassay coefficients of variation were 4.8% and 4.3%, respectively. Hyperleptinaemia was defined according to age-specific and sex-specific serum leptin reference for healthy adolescents as serum leptin >75th centile on the Healthy Lifestyle in Europe by Nutrition in Adolescence (HELENA) Study (8.99 μg/L in males and 39.56 μg/L in females).^25^ These are the only descriptive values for establishing leptin levels in apparently healthy adolescents.

##### Academic performance (AP)

AP was assessed using the student's grades in HS (9th to 12th) and final GPA. Data were collected from administrative records of the Curriculum and Assessment Unit, Ministry of Education (Chile). Since schools may have differed in grading policies, grades (on a scale of 1–7) were transformed into scores (ranging 210–825), following the Ministry of Education criteria. The arithmetic average of each subject taken during each academic year was calculated. Then the result was converted into a score by consulting a conversion table provided by the Department of Assessment, Measurement and Educational Record, University of Chile, which provides specifications on behalf of the Ministry of Education.^26^ The same procedure was used to convert the GPA into a standard score. School grades (9th to 12th) were used as continuous variables, whereas GPA was used as a continuous and categorical variable (GPA scores ≥50th and ≥75th centile in our sample).

##### Anthropometric assessment and weight status at age 16

A trained physician obtained all anthropometric measurements. Weight (kg) and height (m) were assessed with a Seca scale (SECA 703, Seca GmbH and Co. Hamburg, Germany) and a Holtain stadiometer (Harpenden 602 VR, Holtain, Wales, UK) accurate to 0.1 kg and 0.1 cm, respectively. Participants were measured without shoes, wearing underwear, in the Frankfurt position. Body mass index (BMI=(weight (kg)/height (m^2^))) at 16–17 years was calculated, and z-scores and percentiles were estimated according to the 2007 WHO^27^ and Centers for Disease Control and Prevention references, respectively.^28^ Z-scores and percentiles for height at 16–17 years were also calculated. Weight status was defined as: normal weight (BMI z-score from ≥−1 to ≤1 SD), overweight (BMI z-score >1–2 SD) and obesity (BMI z-score >2 SD). Total fat mass was determined on dual X-ray absorptiometry (Lunar Prodigy Corp., Madison, Wisconsin, USA. Software, Lunar iDXA ENCORE 2011, V.13.60.033 Copyright 1998–2010).

##### Insulin sensitivity

Metabolic and hormonal factors, such as glucose and insulin, influence the synthesis and secretion of leptin in the body.^29^ Fasting serum total glucose and insulin levels were performed after a 12-hour overnight fast. Radioimmunoassay (RIA DCP Diagnostic Products Corporation Louisiana, USA. Intra-assay variation ≤5.1%, interassay variation ≤7.1%) was used for insulin determination. Glucose was measured with enzymatic colorimetric test (QCA S.A., Amposta, Spain). Homeostasis model assessment of insulin resistance (HOMA-IR) was calculated as HOMA-IR=(glucose×insulin)/405. HOMA-IR values ≥2.6 were considered insulin resistance (IR), according to national references for healthy adolescents.^30^

##### Diet assessment

Diet has been associated with academic achievement;^31–34^ therefore, it could be a confounder for the association between leptin status and AP. The nutritional quality of items consumed during meals at 16–17 years was measured accounting for the amount of saturated fat, fibre, sugar and salt in the food. We used a validated food frequency questionnaire used in previous studies to assess the usual diet during breakfast, lunch, dinner, snacks at school and snacks at home.^35^ A list of 110 foods and beverages was used. The frequency of food consumption was assessed by a multiple response grid; participants were asked to estimate how often a particular food/beverage was consumed. Categories ranged from ‘never’ to ‘seven times a week’. A software based on the Chilean Food Composition Tables 2010 calculated nutrient intake.^36^ Each meal was considered to be unhealthy (poor nutritional value items, high in fat, sugar, salt and calories), satisfactory (highly processed items although low in fat) or healthy (nutrient rich foods). A score ranging from 0 to 2 was assigned to each meal category, with higher scores representing healthier habits. To estimate the overall quality of diet, scores were summed as a raw score (range 0–10). We applied cut-offs for the Chilean adolescent population to classify the overall diet of participants into three groups: unhealthy (0–4.3), fair (4.4–5.9) and healthy (6–10).^35^

##### Physical activity (PA) habits

We approached PA habits with scheduled, repetitive and planned PA, accounting for the number of weekly hours devoted to school-based physical education (PE) and extracurricular sports. To measure this, we used a questionnaire that was validated in a previous study using accelerometry-based activity monitors in elementary and HS children.^37^ The questionnaire was administered by a researcher to all students at the time they attended the anthropometric examination. Participants were asked: (1) on average, over the past week, how often did you engage in PE? (2) On average, over the past week, how often did you engage in extracurricular sports, either school-organised or non-school-organised? (3) On those days, on average, how long did you engage in such PAs? With this information, we estimated the average hours per week of scheduled PA. Participants having ≤90 min of weekly scheduled PA were considered to be physically inactive.

##### Type of secondary education

In Chile, secondary education includes academic HS, which provide theoretical education in languages, mathematics, history and sciences; vocational school, a combination of theoretical education and vocational training; and adult school, for students who in the past did not receive their secondary education certificate. Data on the type of secondary education attended by participants were retrieved from publicly available records at the Curriculum and Assessment Unit (Ministry of Education).

#### Data from previous waves

##### Parental education

Parental educational attainment provides an important measure of human capital level among populations and also is an important predictor of children's educational outcomes.^38^ In infancy, the participant's mother and father were asked to report the highest schooling level they have been enrolled in, as well as the highest grade they completed at that level. In our analysis, five standard hierarchic levels were defined according to the 2011 International Standard Classification of Education (ISCED): (1) no education completed, (2) first level (primary school or 1st–8th), (3) secondary level (first phase or 9th–10th), (4) secondary level (second phase or 11th–12th), and (5) postsecondary non-tertiary education or short-cycle tertiary education.^39^ Then we merged these categories into two: incomplete secondary education (1+2+3), and complete secondary education or higher (4+5). In health research, parental education has been often used as proxy for socioeconomic background.^40^

##### Iron supplementation in infancy

To control potential design biases, we used a categorical variable denoting whether the participant had received iron supplementation or no added iron at 6–12 months.

### Statistical analysis

Statistical analysis included χ^2^ test for categorical variables and Student's t-test for continuous variables. We tested for effect measure modification (interaction) by sex, weight status and diet in the association between hyperleptinaemia and AP by using two-way analysis of variance. The interactions were non-significant (data not shown), and therefore we did not stratify the analysis. Analysis of covariance (ANCOVA) was used in testing differences in serum leptin concentrations stratified by weight status (normal weight, overweight, obesity). Bonferroni's adjustments for multiple comparisons were used to examine the contrasts between groups. The same technique was used in testing differences in transformed school grades (9th to 12th) and GPA by leptin status (normal serum leptin and hyperleptinaemia). Adjustments were made for sex, weight status and IR status. To examine the association of leptin with school performance, we first conducted linear regression analysis. Serum leptin levels were tested against school grades (9th to 12th and GPA), using two models. Model 1 was adjusted for sex, fat mass and parental education. Model 2 added IR, nutritional quality of diet, PA status, type of secondary education (vocational and adult school), and iron supplementation in infancy (no added iron) to the covariates in model 1. A further analysis was conducted with logistic regressions to estimate the odds of performing ≥50th and ≥75th centile in our sample (outcome) in participants with hyperleptinaemia. For each outcome, three logistic models were estimated. The first one included health-related variables as covariates: weight status, IR, dietary and PA habits as independent variables. A second model added sex, parental education and type of secondary education in which participants completed HS. Finally, Model 3 adjusted the effect of iron supplementation in infancy on academic performance. ORs were estimated along with 95% CI. Data were analysed using Stata for Windows V.12.0 (Lakeway Drive College Station, Texas, USA).

## Results

The mean age of participants during clinical assessments was 16.8 (0.3 SD) years. Males accounted for 51% of the sample. Prevalence of hyperleptinaemia was 14.6% and the mean leptin concentration was 6.0 μg/L in males and 19.4 μg/L in females. Overall, the prevalence of obesity and overweight was 24.6% and 13.5%, respectively. Likewise, 17% of participants had IR. As for school performance, the mean GPA score was 481.1 points (range 269–795), whereas school grades score varied from 471.4 to 494.2 points.

Table 1 shows the descriptive statistics by leptin status at 16–17 years. Mean serum leptin in youths with high leptin levels was 21.4 μg/L in males and 51.3 μg/L in females. In participants with normal serum leptin, levels were 2.2 and 16.0 μg/L for males and females, respectively. Mean BMI z-score and BMI centile at 16–17 years were significantly higher in participants with hyperleptinaemia (p<0.001), and thus the proportion of obese adolescents was significantly higher in this group (43%; p<0.001). Yet 28% of participants with hyperleptinaemia were of normal weight. Other factors did not differ between participants having hyperleptinaemia and those having normal leptin levels. Leptin status was also significantly related to sex.

**Table 1:** Descriptive statistics of participants in the sample

	Total (n=568)		Normal leptin levels (n=485)		Hyperleptinaemia (n=83)		p Value*
	Mean or number	(SD) or percentage	Mean or number	(SD) or percentage	Mean or number	(SD) or percentage	
Chronological age							
Age (years)	16.8†	(0.3)	16.8†	(0.3)	16.8†	(0.3)	NS
Sex							
Male	287	50.5	231	47.6	56	67.5	0.001‡
Female	281	49.5	254	52.4	27	32.5	
Serum leptin							
Males (μg/L)	6.0†	(9.6)	2.2†	(1.9)	21.40†	(13.1)	<0.0001
Females (μg/L)	19.4†	(14.4)	16.0†	(9.7)	51.32†	(12.7)	<0.0001
Anthropometrics							
Height at 16 years (WHO z-score)	−0.45†	0.8	−0.47†	0.8	−0.35†	0.8	NS
Height at 16 years (CDC percentile)	35.0†	24.7	34.5†	24.7	37.8†	24.2	NS
BMI at 16 years (WHO z-score)	0.63†	(1.2)	0.47†	(1.1)	1.58†	(1.3)	<0.0001
BMI at 16 years (CDC percentile)	64.4†	28.0	61.4†	27.4	81.4†	25.3	<0.0001
Weight status							
Normal weight	352	61.9	329	67.8	23	27.7	<0.0001
Overweight	139	24.5	115	23.7	24	28.9	
Obesity	77	13.6	41	8.5	26	43.4	
Insulin sensitivity							
HOMA-IR	1.77†	(1.2)	1.64†	(1.0)	2.39†	(1.9)	<0.0001
IR	83	14.6	59	12.5	24	25.3	<0.0001‡
PA patterns							
Weekly scheduled PA≤90 min	322	56.7	275	56.7	47	56.6	NS‡
Diet habits							
Unhealthy diet	162	28.5	137	28.3	25	30.1	NS‡
Parental education							
Mother's schooling: incomplete secondary	377	34.0	168	34.6	25	30.1	NS‡
Father's schooling: incomplete secondary	159	28.0	140	28.9	19	22.9	NS‡
Type of secondary education							
Adult school	98	17.3	79	16.3	19	22.9	NS‡
Iron supplementation (infancy)							
Non-added Fe	238	41.9	205	42.3	33	39.8	NS‡

Normal weight: BMI z-score ≤1 SD; overweight: BMI z-score >1 and ≤2 SD; obesity: BMI z-score >2 SD.

Hyperleptinaemia defined according to the cut-offs published by Köster-Weber *et al*.

*Student's t-test, except as indicated.

†Values expressed as mean and (SD). Otherwise, values are number of observations and percentage.

‡χ^2^ test (Pearson).

BMI, body mass index; CDC, Centers for Disease Control and Prevention; HOMA, homeostasis model assessment of IR; IR, insulin resistance; NS, not significant; PA, physical activity.

**Figure 1:**
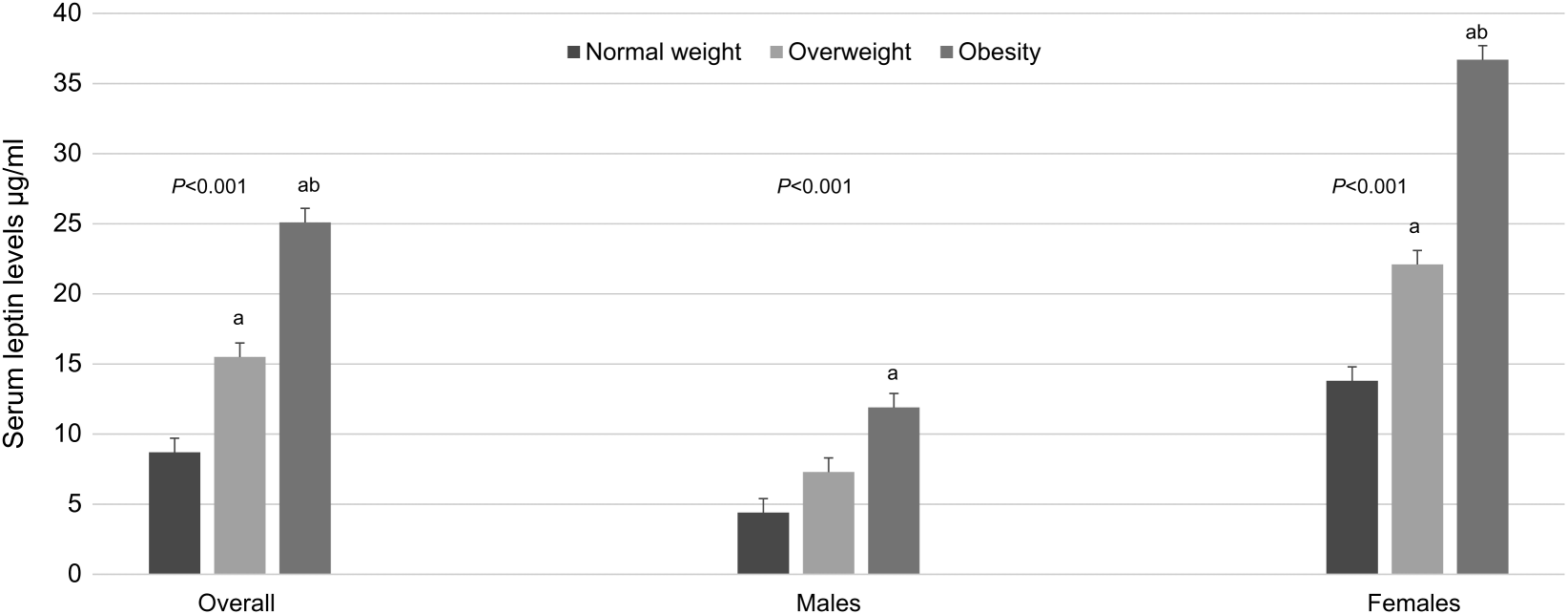
Mean values of serum leptin in adolescents in the sample, by weight status (n=568). Analysis of covariance (ANCOVA) was used in testing differences in serum leptin concentrations stratified by weight status (normal weight, overweight, obesity). Bonferroni's adjustments for multiple comparisons were used to examine the contrasts between the groups. (a) Significantly different from the normal weight group. (b) Significantly different from the overweight group. Normal weight: Body mass index (BMI) z-score from −1 to 1 SD. Overweight: BMI z-score from >1 to 2 SD. Obesity: BMI z-score from >2 SD. Mean values are shown with error bars representing SE.

Overall in the sample, significant contrasts in serum leptin concentrations were seen when comparing normal weight participants with overweight (p<0.001) and obese (p<0.001) ones, and also when comparing overweight adolescents with those being obese (p<0.001). In males, serum leptin levels were significantly higher in obese participants compared with their normal weight peers (p=0.01). Last, differences in serum leptin levels were significant when comparing females of healthy weight with females who were overweight (p<0.001) and obese (p<0.001). Also, significant contrasts in serum leptin concentrations were found when comparing overweight females with obese females (p<0.001) (figure 1).

**Figure 2:**
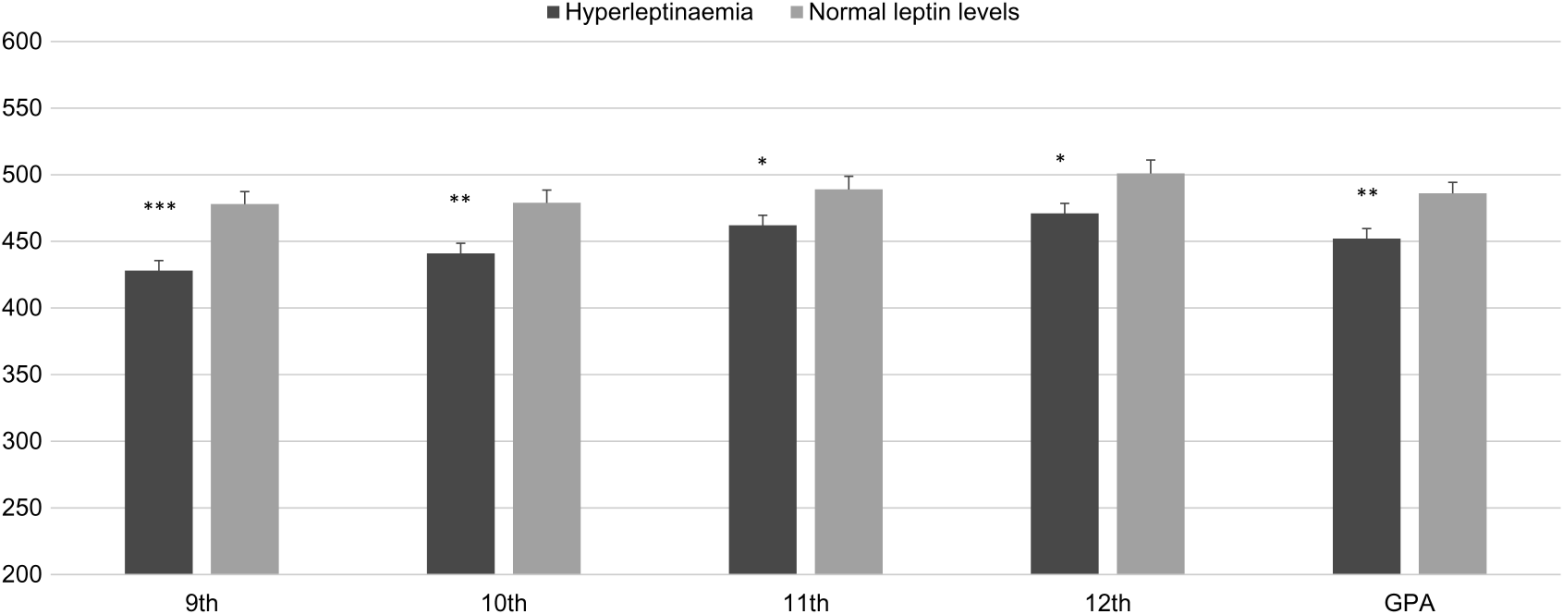
High-school grades and final grade point average (GPA) in Chilean youths by leptin status at age 16–17 (n=568). Differences in school performance by leptin status in adolescence. Grades and GPA expressed as standardised scores according to the Chilean Ministry of Education. Hyperleptinaemia defined according to the cut-offs published by Köster-Weber *et al*. Adjustments were made for sex, weight status (overweight and obesity) and insulin sensitivity (homeostasis model assessment of insulin resistance >2.6) at 16–17 years. Mean values are shown with error bars representing SE. Significance: *p<0.05; **p<0.01; ***p<0.001.

As for academic outcomes, youths with hyperleptinaemia had significantly lower school grades and GPA compared with participants with normal serum leptin (table 2 and figure 2). After adjusting for sex, weight status and insulin sensitivity, ANCOVA showed that the grades mean difference varied from 28.1 points in 11th grade (p=0.038) to 38.6 points in 9th grade (p=0.002), whereas GPA mean difference was 33.8 points (p=0.004).

**Table 2:** Association of academic performance across HS grades with leptin status at 16–17 years (n=568)

	Hyperleptinaemia (n=83)	Normal leptin levels (n=485)	Mean score difference			
HS grade level	Mean score	Mean score		(95% CI)	*t*	p Value
9th	437.6	476.2	−38.6	−63.6 to −13.6	−3.04	0.002
10th	446.7	478.1	−31.4	−157.5 to −5.32	−2.36	0.018
11th	456.3	484.4	−28.1	−54.6 to −1.52	−2.08	0.038
12th	470.3	500.1	−29.8	−57.2 to −2.48	−2.14	0.033
Final GPA	454.9	488.7	−33.8	−56.9 to −10.7	−2.88	0.004

Grades (9th to 12th) and final GPA expressed as scores, according to the Chilean Ministry of Education.

Hyperleptinaemia defined according to the cut-offs published by Köster-Weber *et al*.

Adjustments were made for weight status and insulin sensitivity at 16–17 years.

GPA, grade point average; HS, high school.

We observed a negative and significant association of serum leptin levels at 16–17 years with AP, as measured by transformed school grades (table 3). Serum leptin was negatively correlated with school performance in 9th, 10th and 12th grades, and, similarly, it was negatively correlated with HS GPA. For instance, for a one-unit increase in leptin levels, GPA decreased 0.80 points. School grades in 11th also decreased with leptin rise, but the association was non-significant in our sample.

**Table 3:** Regression coefficients of the association between serum leptin levels at 16–17 years and academic performance in HS

	Intercept	Coefficient	Robust SE	R^2^
9th grade				
Model 1	491.2***	−0.56	0.32	0.04
Model 2	501.9***	−0.62*	0.30	0.09
10th grade				
Model 1	495.6***	−0.76	0.43	0.02
Model 2	508.3***	−0.82*	0.37	0.14
11th grade				
Model 1	511.7***	−0.47	0.35	0.04
Model 2	522.0***	−0.57	0.34	0.10
12th grade				
Model 1	527.9***	−0.87*	0.37	0.04
Model 2	540.5***	−0.98***	0.37	0.13
HS GPA				
Model 1	511.2***	−0.71*	0.31	0.04
Model 2	521.3***	−0.80***	0.30	0.16

SE are robust to heteroscedasticity.

Model 1: adjusted for sex, fat mass and parental education. Model 2: also included IR, quality of diet, PA status, type of secondary education and iron supplementation in infancy.

*p<0.05; **p<0.01; ***p<0.001.

GPA, grade point average; HS, high school; PA, physical activity.

Table 4 contains the estimated association between having a final GPA≥50th centile in the sample and leptin status at 16 years after controlling for other influences. After full adjustments (model 3), the odds of performing ≥50th centile in youths with hyperleptinaemia were 56% (95% CI 0.32% to 0.95%) that of their peers with normal serum leptin. We likewise found that performing ≥50th centile was negative and significantly associated with unhealthy dietary habits (OR 0.52; 95% CI 0.33 to 0.81), being male (OR 0.63; 95% CI 0.28 to 0.80) and attending adult schools (OR 0.38; 95% CI 0.23 to 0.62). Association of academic results with weight status, IR and parental schooling was non-significant at an α level of 0.05. Iron supplementation in infancy was not associated with GPA later in adolescence.

**Table 4:** Relationship between having a GPA>50th centile and leptin resistance in Chilean youths after controlling for other health, sociodemographic and educational influences (n=568)

	Model 1		Model 2		Model 3	
	OR	95% CI	OR	95% CI	OR	95% CI
Hyperleptinaemia	0.50**	0.30 to 0.84	0.50*	0.33 to 0.94	0.56*	0.32 to 0.95
Overweight	0.87	0.57 to 1.30	0.70	0.38 to 1.27	0.65	0.35 to 1.22
Obesity	0.99	0.86 to 1.18	0.86	0.49 to 1.50	0.81	0.45 to 1.33
IR	0.94	0.80 to 1.11	0.93	0.49 to 1.09	0.78	0.55 to 1.18
Unhealthy diet	(…)		0.53	0.34 to 0.81	0.52**	0.33 to 0.81
Physically inactive	(…)		0.89	0.59 to 1.34	0.99	0.64 to 1.53
Male sex	(…)		(…)		0.63**	0.28 to 0.80
Maternal education: incomplete HS	(…)		(…)		1.09	0.82 to 1.74
Paternal education: incomplete HS	(…)		(…)		0.96	0.65 to 1.45
Adult HS	(…)		(…)		0.38***	0.23 to 0.62
No Fe supplement (infancy)	(…)		(…)		0.91	0.64 to 1.28

(…) Non-observed.

Significance level: *p<0.05; **p<0.01; ***p<0.001.

Hyperleptinaemia defined according to the cut-offs published by Köster-Weber *et al*.

Overweight: BMI z-score >1 and ≥2 SD; obesity: BMI z-score ≥2 SD.

IR: HOMA-IR 2.6.

Unhealthy diet: diet high in simple carbohydrates and saturated fats.

Physically inactive: scheduled PA≤90 min/week.

Adult HS: education for students who in the past were unable to receive their diploma.

BMI, body mass index; GPA, grade point average; HOMA, homeostasis model assessment of IR; HS, high school; IR, insulin resistance; PA, physical activity.

Table 5 shows the association between having a final GPA≥75th centile in the sample and leptin status at 16 years after controlling for a number of confounders. In a fully adjusted model (model 3), the likelihood of performing ≥75th centile in leptin resistant youths was 42% (95% CI 0.19 to 0.89) that of students with normal leptin levels. We likewise found that school performance was negatively related to being male (OR 0.43; 95% CI 0.28 to 0.71), having unhealthy dietary habits (OR 0.41; 95% CI 0.24 to 0.75), father's schooling (incomplete secondary education) (OR 0.57; 95% CI 0.35 to 0.93) and the type of secondary education (OR 0.35; 95% CI 0.18 to 0.69). Again, weight status, IR and maternal educational level were unrelated to school performance. Iron supplementation in infancy was not related to HS performance as measured by having a GPA in the highest quartile.

**Table 5:** Relationship between having a GPA>75th centile and leptin resistance in Chilean youths after controlling for other health, sociodemographic and educational influences (n=568)

	Model 1		Model 2		Model 3	
	OR	95% CI	OR	95% CI	OR	95% CI
Hyperleptinaemia	0.35***	0.17 to 0.72	0.35***	0.17 to 0.73	0.42*	0.19 to 0.89
Overweight	0.66	0.40 to 1.10	0.67	0.42 to 1.13	0.62	0.37 to 1.05
Obesity	0.83	0.42 to 1.64	0.83	0.42 to 1.64	0.74	0.36 to 1.54
IR	0.79	0.41 to 0.95	0.69	0.44 to 1.06	0.73	0.46 to 1.13
Unhealthy diet	(…)		0.43***	0.26 to 0.78	0.41***	0.24 to 0.75
Physically inactive	(…)		1.01	0.63 to 1.59	1.00	0.66 to 1.54
Male sex	(…)		(…)		0.43***	0.28 to 0.71
Maternal education: incomplete HS	(…)		(…)		1.07	0.68 to 1.67
Paternal education: incomplete HS	(…)		(…)		0.57*	0.35 to 0.93
Adult HS	(…)		(…)		0.35***	0.18 to 0.69
No Fe supplement (infancy)	(…)		(…)		0.71	0.48 to 1.06

(…) Non-observed.

Significance level: *p<0.05; **p<0.01; ***p<0.001.

Hyperleptinaemia defined according to the cut-offs published by Köster-Weber *et al*.

Overweight: BMI z-score >1 and ≥2 SD; obesity: BMI z-score ≥2 SD.

IR: HOMA-IR 2.6.

Unhealthy diet: diet high in simple carbohydrates and saturated fats.

Physically inactive: scheduled PA≤90 min/week.

Adult HS: education for students who in the past were unable to receive their diploma.

BMI, body mass index; GPA, grade point average; HOMA, homeostasis model assessment of IR; HS, high school; IR, insulin resistance; PA, physical activity.

## Discussion

In a sample of HS graduates, we examined the relationship between leptin status and cognition by using functional measures such as HS grades and GPA. To the best of our knowledge, this is the first study addressing the link between leptin status and this domain of cognition in a younger-age human population. Compared with students having normal serum leptin, those having hyperleptinaemia had lower school grades and GPA. Similarly, they had lowers odds of performing ≥50th and ≥75th centile of the sample. Even after controlling for relevant confounders, the association between leptin status in adolescence and AP remained significant.

Our results are of importance for several reasons. Leptin plays a key role in memory processing through induction of hippocampal and prefrontal cortex synaptic plasticity. However, a growing body of evidence suggests that elevated plasma leptin levels may limit the potential for synaptic plasticity and could partially explain some cognitive deficits.^3^ ^7^ ^17–20^ Likewise, links are strong between memory performance and academic outcomes. In children, working memory and particularly the ability to retrieve and manipulate information from long-term memory has been found to predict math, reading and spelling outcomes, even after controlling for IQ.^21^ Dysfunctions in this domain can lead to learning difficulties in activities that involve storing and processing information.^21^ ^22^ Furthermore, HS grades predict higher education outcomes and subsequent job status and income.^41^

Very few studies have explored the link between leptin and cognition in younger animal populations, and their findings are in line with ours. Oomura *et al*^17^ showed that leptin modulates higher neural functions in mice aged 4–8 weeks. While infusion of low doses of leptin enhanced learning and memory performance and hippocampal LTP, high doses impaired them. The notion that hyperleptinaemia could be responsible for some cognitive deficits in adolescent mice is supported by Valladolid-Acebes *et al*.^18–20^ In age-matched mice, short-term exposure to high-fat diet compromised hippocampal dependent learning and memory. Moreover, in adolescent mice, the behavioural impairment was accompanied by changes in hippocampal morphology and functionality of leptin receptors within the hippocampus.

Leptin levels in this Chilean sample were lower than those reported for European adolescents,^25^ but higher than those reported for healthy adolescents in other Latin America countries and Asia.^42^ ^43^ Population differences in the epidemiological and nutrition transition may in part explain the disparity in levels of circulating leptin. Increased intake of fat, sugar and processed foods, reduced PA, and increased risk of non-communicable disease, including obesity, are more prevalent in the last stages of the transition, which is the case of Chile and the European countries.^44^

Although an association between weight status and academic results was not observed in this sample, obesity in paediatric populations has been related to impaired cognitive function^45^ ^46^ and the ability to perform well in school.^47^ ^48^ Some authors have postulated that cognitive impairment may actually precede excessive weight gain.^18^ ^49–51^ Two main ideas are behind this view: first, impairment of learning is observed before other metabolic alterations; second, while the adverse effects of consuming high fat/high-sugar diet on cognitive function is a good predictor of subsequent weight gain, the effect of those nutrients on weight gain does not reliably predict subsequent cognitive deficit. Our results suggest that leptin might mediate, in part, the effect of weight status on cognition and academic outcomes.

It is very likely that hyperleptinaemia in most individuals in our sample may lead to both under-responsiveness to exogenous leptin and impairment of signal transduction in target neurons. Although obesity has been traditionally associated with hyperleptinaemia in the non-elderly population, new data indicate that fructose, sucrose and triglycerides may induce hyperleptinaemia regardless of the amount of body fat.^15^ In our sample, less than half of the participants having hyperleptinaemia were obese, and 28% were of normal weight. Likewise, almost 80% had a diet in the intermediate or unhealthy zone, which means excessive intake of refined sugars and fats. Administration of even small quantities of triglycerides to normal weight rats immediately resulted in dysfunctional leptin transport across the BBB.^52^ ^53^ Similarly, rats exposed to high fructose diet demonstrated an impaired response to peripheral leptin injection and central leptin infusion. However, switching to a sugar-free diet improved leptin sensitivity.^54^ In both cases, chronic fructose and triglycerides consumption led to impaired responsiveness to exogenous leptin prior to body weight.

Our findings also confirm the role of nutrition in academic outcomes. Participants having an unhealthy diet had significantly lower odds of performing well in HS, independent of leptin status. Numerous studies report that diet relates to specific outcomes that are important for the educational attainment of children and adolescents.^31–34^ The consumption of refined carbohydrates and saturated fats is linked to impaired cognitive performance. Exposure to these macronutrients interferes directly with hippocampal functioning by cutting down the production of neurotrophins, increasing neuroinflammatory markers, and altering the BBB.^50^ ^55^ In addition, indirect adverse effects of diet on cognitive functioning, including reduced potential for synaptic plasticity and trafficking of neurotransmitter receptors in the hippocampus, have been related to diet-induced leptin and IR.^18–20^ ^49^ ^50^

Last, in our sample, the share of participants with hyperleptinaemia was significantly higher in males compared with females. Notwithstanding that leptin is produced in the fat tissue, serum leptin concentration is also dependent on determinants such as insulin sensitivity and overconsumption of sucrose and fructose. Male and female participants in our study had similar prevalence of excess weight and IR, but the proportion of adolescents having a diet high in simple sugars, mostly sucrose and fructose from candies and sugar sweetened beverages (a dietary pattern regarded as a fair diet in our questionnaire), was significantly higher among males compared with females. Population surveys in the Chilean adolescent population confirm that adolescent males have excess consumption of simple carbohydrates.^56^ ^57^

### Implications from these results

The Chilean adolescent population is highly exposed to risk factors for hyperleptinaemia. As reported by the 2014 Chilean National Food Consumption Survey,^56^ dietary habits of poor nutritional quality are widespread. Adolescents aged 14–18 rank first in the consumption of refined sugar (121 g/day) and second in the consumption of saturated fats. According to this survey, the prevalence of overweight and obesity in adolescents is 38% and 13%, respectively.

A further implication from this study relates the fact that exposure to abnormally high levels of leptin would be starting early in life. A non-modifiable condition such as ageing is associated with hyperleptinaemia, but exposure to high fat/high-sugar diet as well as to overweight and obesity are avoidable. While schools should serve as a point of entry for the promotion of healthy lifestyles, the food industry uses schools as a means of reaching young consumers. Despite the efforts to limit the availability of unhealthy foods in schools, in the Latin-American Southern Cone 70% of adolescents drink sugar-sweetened beverages on a daily basis.^57^ Frequent (1–2 times/week) and very frequent (≥3 times/week) fast-food consumption among Latin American adolescents ranges from 40% in Uruguay to 70% in Bolivia, according to a recent international study.^58^

The following points should be considered in the design and application of preventive strategies. Fructose-induced hyperleptinaemia may be reversible, which means that there is a possibility for improvement. Second, hyperleptinaemia may be triggered by exposure to even small amounts of saturated fats and refined sugars, which are major components of the Western diet. Moreover, the effects on leptin levels appear soon after exposure to these macronutrients. Finally, hyperleptinaemia is a precondition for LR; at this point, it might be late for adolescents to reach their full cognitive and academic potential.

### Study limitations and strengths

Our results support the notion that leptin has an important role in cognitive function in addition to its role in energy balance regulation. A further strength is that we approached this topic in adolescents, whereas previous studies have been conducted in adults or the elderly. Third, we used functional measures of cognition (eg, AP in HS), aiming to examine this relation in the ‘real’ world. In spite of these strengths, the study has limitations that should be considered when interpreting the findings. First, our sample is not representative of the Chilean adolescent population, as it consists of adolescents from low-to-middle SES. However, SES level may be especially important. The prevalence of unhealthy dietary habits and obesity, both of which may lead to hyperleptinaemia, is higher among adolescents from low-to-middle SES compared with adolescents of high SES.^56^ Second, we used cut-offs for hyperleptinaemia defined on the basis of statistical criteria in a European cohort, which are the only values described for healthy adolescents. It is worth noting that Chile, like Western European countries, is in the last stage of the epidemiological transition. Yet future studies should use cut-offs based on biological risk. Third, owing to data constraints, the prevalence of neuropsychiatric conditions was not included in the study. Leptin effects on memory and cognition might be confounded by the coexistence of neuropsychiatric disorders such as depression, schizophrenia and substance-related disorders. Fourth, we did not consider the mediating effect of potential learning and cognitive disorders which may impact student's academic functioning. Also, information on family structure is lacking in the analysis. Yet we considered the impact of parental education. Since association is not enough to prove causation, future studies should replicate and extend this analysis in other young populations, and further investigate how leptin influences cognitive and academic outcomes.

## References

[1] Banks WA Extrahypothalamic effects of leptin: a therapeutic for depression and dementia? Endocrinology 2011;152:2539–41. 10.1210/en.2011-1161 21697453

[2] Chowen J, Argente J Leptin and the brain. Horm Mol Biol Clin Invest 2011;7:351–60. 10.1515/HMBCI.2011.113 25961273

[3] Irving AJ, Harvey J Leptin regulation of hippocampal synaptic function in health and disease. Philos Trans R Soc Lond B Biol Sci 2013;369:20130155 10.1098/rstb.2013.0155 24298156 PMC3843886

[4] Harvey J, Shanley LJ, O'Malley D Leptin: a potential cognitive enhancer? Biochem Soc Trans 2005;33(Pt 5):1029–32. 10.1042/BST20051029 16246038

[5] Harvey J, Solovyova N, Irving A Leptin and its role in hippocampal synaptic plasticity. Prog Lipid Res 2006;45:369–78. 10.1016/j.plipres.2006.03.001 16678906 PMC1762032

[6] Harvey J Leptin regulation of neuronal morphology and hippocampal synaptic function. Front Synaptic Neurosci 2013;5:3 10.3389/fnsyn.2013.00003 23964236 PMC3734345

[7] Farr SA, Banks WA, Morley JE Effects of leptin on memory processing. Peptides 2006;27:1420–5. 10.1016/j.peptides.2005.10.006 16293343

[8] Val-Laillet D, Layec S, Guerin S Changes in brain activity after a diet-induced obesity. Obesity (Silver Spring) 2011;19:749–56. 10.1038/oby.2010.292 21212769

[9] Yu Y, Wu Y, Szabo A Teasaponin improves leptin sensitivity in the prefrontal cortex of obese mice. Mol Nutr Food Res 2015;59:2371–82. 10.1002/mnfr.201500205 26314570

[10] Zlokovic BV, Jovanovic S, Miao W Differential regulation of leptin transport by the choroid plexus and blood-brain barrier and high affinity transport systems for entry into hypothalamus and across the blood-cerebrospinal fluid barrier. Endocrinology 2000;141:1434–41. 10.1210/endo.141.4.7435 10746647

[11] Knight ZA, Hannan KS, Greenberg ML Hyperleptinaemia is required for the development of leptin resistance. PLoS ONE 2010;5:e11376 10.1371/journal.pone.0011376 20613882 PMC2894068

[12] Scarpace PJ, Zhang Y Elevated leptin: consequence or cause of obesity? Front Biosci 2007;12:3531–44. 10.2741/2332 17485319

[13] Morris D, Rui L Recent advances in understanding leptin signaling and leptin resistance. Am J Physiol Endocrinol Metab 2009;297:E1247–59. 10.1152/ajpendo.00274.2009 19724019 PMC2793049

[14] Zhou Y, Rui L Leptin signaling and leptin resistance. Front Med 2013;7:207–22. 10.1007/s11684-013-0263-5 23580174 PMC4069066

[15] Vasselli JR, Scarpace PJ, Harris RB Dietary components in the development of leptin resistance. Adv Nutr 2013;4:164–75. 10.3945/an.112.003152 23493533 PMC3649097

[16] Fam BC, Morris MJ, Hansen MJ Modulation of central leptin sensitivity and energy balance in a rat model of diet-induced obesity. Diabetes Obes Metab 2007;9:840–52. 10.1111/j.1463-1326.2006.00653.x 17924866

[17] Oomura Y, Hori N, Shiraishi T Leptin facilitates learning and memory performance and enhances hippocampal CA1 long-term potentiation and CaMK II phosphorylation in rats. Peptides 2006;27:2738–49. 10.1016/j.peptides.2006.07.001 16914228

[18] Valladolid-Acebes I, Stucchi P, Cano V High-fat diets impair spatial learning in the radial-arm maze in mice. Neurobiol Learn Mem 2011;95:80–5. 10.1016/j.nlm.2010.11.007 21093599

[19] Valladolid-Acebes I, Merino B, Principato A High-fat diets induce changes in hippocampal glutamate metabolism and neurotransmission. Am J Physiol Endocrinol Metab 2012;302 (4):E396–402. 10.1152/ajpendo.00343.2011 22114023

[20] Valladolid-Acebes I, Fole A, Martín M Spatial memory impairment and changes in hippocampal morphology are triggered by high-fat diets in adolescent mice. Is there a role of leptin? Neurobiol Learn Mem 2013;106:18–25. 10.1016/j.nlm.2013.06.012 23820496

[21] Alloway TP, Alloway RG Investigating the predictive role of working memory and IQ in academic attainment. J Exp Child Psychol 2010;106:20–9. 10.1016/j.jecp.2009.11.003 20018296

[22] McClellan MM, Cameron CE, Duncan R Predictors of early growth in academic achievement: the head-toes-knees-shoulders task. Front Psychol 2014;5:599 10.3389/fpsyg.2014.00599 25071619 PMC4060410

[23] Lozoff B, Castillo M, Clark KM Iron supplementation in infancy contributes to more adaptive behavior at 10 years of age. J Nutr 2014;144:838–45. 10.3945/jn.113.182048 24717366 PMC4018948

[24] Burrows R, Correa-Burrows P, Reyes M High cardiometabolic risk in healthy Chilean adolescents: associations with anthropometric, biological and lifestyle factors. Public Health Nutr 2016;19:486–93. 10.1017/S1368980015001585 25990645 PMC4654715

[25] Köster-Weber T, Valtueña J, Breidenassel C Reference values for leptin, cortisol, insulin and glucose, among European adolescents and their association with adiposity: the HELENA Study. Nutr Hosp 2014;30:1181–90. 10.3305/nh.2014.30.5.7982 25365025

[26] *High-school grades conversion table*. College Admission process for the academic year 2014. Department of Assessment, Measurement and Educational Record, University of Chile 2013 http://www.psu.demre.cl/proceso-admision/factores-seleccion/tabla-transformacion-nem

[27] de Onis M, Onyango AW, Borghi E Development of a WHO growth reference for school-aged children and adolescents. Bull World Health Organ 2007;85:660–7. 10.2471/BLT.07.043497 18026621 PMC2636412

[28] Fryar CD, Gu Q, Ogden CL Anthropometric reference data for children and adults: United States, 2007–2010. Vital Health Stat 11 2012;11:1–48. 25204692

[29] Fried SK, Ricci MR, Russell CD Regulation of leptin production in humans. J Nutr 2000;130:3127S–31S. 11110887 10.1093/jn/130.12.3127S

[30] Burrows R, Correa-Burrows P, Reyes M Healthy Chilean adolescents with HOMA-IR ≥ 2.6 have increased cardiometabolic risk: association with genetic, biological, and environmental factors. J Diabetes Res 2015;2015:783296 10.1155/2015/783296 26273675 PMC4530255

[31] Feinstein L, Sabates R, Sorhaindo A Dietary patterns related to attainment in school: the importance of early eating patterns. J Epidemiol Comm Health 2008;62:734–9. 10.1136/jech.2007.068213 18621960

[32] Kristjánsson AL, Sigfúsdóttir ID, Allegrante JP Health behavior and academic achievement among adolescents: the relative contribution of dietary habits, physical activity, body mass index, and self-esteem. Health Educ Behav 2010;37:51–64. 10.1177/1090198107313481 18541647

[33] Øverby NC, Lüdemann E, Høigaard R Self-reported learning difficulties and dietary intake in Norwegian adolescents. Scand J Pub Health 2013;41:754–60. 10.1177/1403494813487449 23676256

[34] Correa-Burrows P, Burrows R, Blanco E Nutritional quality of diet and academic performance in Chilean students. Bull World Health Organ 2016;94:185–92. 10.2471/BLT.15.161315 26966329 PMC4773934

[35] Burrows AR, Díaz BE, Sciaraffia MV [Dietary intake and physical activity in school age children]. Rev Med Chil 2008;136:53–63. doi:/S0034-98872008000100007 18483654

[36] Tablas Chilenas de Composición Química de los Alimentos. Santiago de Chile: Ministerio de Salud 2010 http://web.minsal.cl/composicion_alimentos

[37] Godard MC, Rodríguez N Mdel P, Díaz N [Value of a clinical test for assessing physical activity in children]. Rev Med Chil 2008;136:1155–62. doi:/S0034-98872008000900010 19030660

[38] Dobow EF, Boxer P, Huesmann LR Long-term effects of parents’ education on children's educational and occupational success: mediation by family interactions, child aggression, and teenage aspirations. Merrill Palmer Q (Wayne State Univ Press) 2009;55:224–49. 10.1353/mpq.0.0030 20390050 PMC2853053

[39] International Standard Classification of Education. ISCED 2011. UNESCO Institute for Statistics Paris: United Nations Educational, Scientific and Cultural Organization (UNESCO), 2012 http://www.uis.unesco.org/Education/Documents/isced-2011-en.pdf

[40] Braveman P, Cubbin C, Egerter S Socioeconomic status in health research: one size does not fit all. JAMA 2005;294:2879–88. 16352796 10.1001/jama.294.22.2879

[41] French M, Homer J, Popovic I What you do in high-school matters: high-school GPA, educational attainment, and labor market earnings as a young adult. East Econ J 2015;41:370–86.

[42] Brandão CM, Lombardi MT, Nishida SK Serum leptin concentration during puberty in healthy non-obese adolescents. Braz J Med Biol Res 2003;36:1293–6. 10.1590/S0100-879X2003001000003 14502359

[43] Xi H, Zhang L, Guo Z Serum leptin concentration and its effect on puberty in Naqu Tibetan adolescents. J Physiol Anthropol 2011;30:111–17. 10.2114/jpa2.30.111 21636954

[44] Popkin BM, Adair LS, Ng SW Global nutrition transition and the pandemic of obesity in developing countries. Nutr Rev 2012;70:3–21. 10.1111/j.1753-4887.2011.00456.x 22221213 PMC3257829

[45] Reyes S, Peirano P, Peigneux P Inhibitory control in otherwise healthy overweight 10-year-old children. Int J Obes (Lond) 2015;39:1230–5. 10.1038/ijo.2015.49 25869603 PMC4526395

[46] Groppe K, Elsner B. Executive function and weight status in children: A one-year longitudinal perspective. Child Neuropsychol 2015. [Epub ahead of print 29 Sep 2015]. 10.1080/09297049.2015.1089981 26416504

[47] Kamijo K, Khan NA, Pontifex MB The relation of adiposity to cognitive control and scholastic achievement in preadolescent children. Obesity (Silver Spring) 2012;20:2406–11. 10.1038/oby.2012.112 22546743 PMC3414677

[48] Carey FR, Singh GK, Brown HS III Educational outcomes associated with childhood obesity in the United States: cross-sectional results from the 2011–2012 National Survey of Children's Health. Int J Behav Nutr Phys Act 2015;12(Suppl 1):S3 10.1186/1479-5868-12-S1-S3 26222699 PMC4663995

[49] Kanoski SE, Davidson TL Different patterns of memory impairments accompany short- and longer-term maintenance on a high-energy diet. J Exp Psychol Anim Behav Process 2010;36:313–19. 10.1037/a0017228 20384410

[50] Davidson TL, Hargrave SL, Swithers SE Inter-relationships among diet, obesity and hippocampal-dependent cognitive function. Neuroscience 2013;253:110–22. 10.1016/j.neuroscience.2013.08.044 23999121 PMC3934926

[51] Zhang Z, Manson KF, Schiller D Impaired associative learning with food rewards in obese women. Curr Biol 2014;24:1731–6. 10.1016/j.cub.2014.05.075 25042588

[52] Banks WA, Coon AB, Robinson SM Triglycerides induce leptin resistance at the blood-brain barrier. Diabetes 2004;53:1253–60. 10.2337/diabetes.53.5.1253 15111494

[53] Vasselli JR, Spilka M Circulating triglycerides and leptin resistance: a test of the triglyceride hypothesis. Obesity 2010;18:S78.

[54] Shapiro A, Mu W, Roncal C Fructose-induced leptin resistance exacerbates weight gain in response to subsequent high-fat feeding. Am J Physiol Regul Integr Comp Physiol 2008;295:R1370–5. 10.1152/ajpregu.00195.2008 18703413 PMC2584858

[55] Gómez-Pinilla F Brain foods: the effects of nutrients on brain function. Nat Rev Neurosci 2008;9:568–78. 10.1038/nrn2421 18568016 PMC2805706

[56] Encuesta Nacional de Consumo Alimentario*.* Informe final. Santiago: Ministry of Health, 2014.

[57] Encuesta Global de Salud Escolar (Chile). Unidad de epidemiología. Santiago: Ministry of Health, 2013.

[58] Braithwaite I, Stewart AW, Hancox RJ, *et al*. ISAAC Phase Three Study Group. Fast-food consumption and body mass index in children and adolescents: an international cross-sectional study. *BMJ Open* 2014;4:e005813. 10.1136/bmjopen-2014-005813 PMC4265088 25488096

